# Effects of perioperative cognitive function training on postoperative cognitive dysfunction and postoperative delirium: a systematic review and meta-analysis

**DOI:** 10.3389/fneur.2023.1146164

**Published:** 2023-06-21

**Authors:** Li Zhao, Hongyu Zhu, Wei Mao, Xuelei Zhou, Ying Xie, Linji Li

**Affiliations:** Department of Anesthesiology, The Second Clinical Medical College, North Sichuan Medical College, Nanchong Central Hospital, Nanchong, China

**Keywords:** cognitive function training, cognitive intervention, perioperative cognitive disorders, postoperative cognitive dysfunction, postoperative delirium

## Abstract

**Background:**

Randomized controlled trials (RCTs) have shown conflicting results regarding the effects of perioperative cognitive training (CT) on the incidence of postoperative cognitive dysfunction (POCD) and postoperative delirium (POD). We, therefore, performed a meta-analysis to assess the overall effects of studies on this topic.

**Methods:**

We searched PubMed, Embase, the Cochrane Library, and Web of Science for all RCTs and cohort studies that investigated the effects of perioperative CT on the incidence of POCD and POD. Data extraction and quality assessment were conducted independently by two researchers.

**Results:**

This study included nine clinical trials with a total of 975 patients. The results showed that perioperative CT significantly reduced the incidence of POCD compared with the control group [risk ratio (RR) = 0.5, 95% CI (confidence interval): 0.28–0.89, *P* = 0.02]. Nevertheless, for the incidence of POD, the difference between the two groups was not statistically significant (RR = 0.64; 95% CI: 0.29–1.43, *P* = 0.28). In addition, the CT group had less postoperative decline in the cognitive function scores compared with the control group [mean differences (MD): 1.58, 95% CI: 0.57–2.59, *P* = 0.002]. In addition, there were no statistically differences in length of hospital stay between the two groups (MD: −0.18, 95% CI: −0.93–0.57, *P* = 0.64). Regarding CT adherence, the proportion of patients in the cognitive training group who completed the planned duration of CT was 10% (95% CI: 0.05–0.14, *P* = 0.258).

**Conclusion:**

Our meta-analysis revealed that perioperative cognitive training is possibly an effective measure to reduce the incidence of POCD, but not for the incidence of POD.

**Systematic review registration:**

https://www.crd.york.ac.uk/prospero/display_record.php?ID=CRD42022371306, identifier: CRD42022371306.

## 1. Introduction

Alongside an aging population, the number of older adults undergoing surgical procedures is also increasing (1). Postoperative cognitive dysfunction (POCD) and postoperative delirium (POD) are common and serious postoperative complications in older people that can prolong hospital stay, reduce the quality of life, increase healthcare costs, and even increase mortality (2, 3). POCD is defined as a significant reduction in the cognitive performance from baseline following surgery (4). The incidence of POCD reportedly varies from 1.5 to 28% (5). POD is a postoperative acute and reversible cerebral dysfunction, mainly manifested as confusion and altered consciousness (6). Studies have reported that the incidence of POD after cardiovascular surgery is as high as 15.3–23.4% (7). The specific mechanisms of POCD and POD are still unclear, but studies have revealed that POCD and POD are the result of the interaction between multiple risk factors, including the patient's cognitive function level, coexisting chronic diseases, nutritional status, use of anesthetic drugs, surgery, and pain (8–12). Because of the difficulty in the prevention and treatment of POCD and POD, it is important to find an effective method to reduce the incidence of POCD and POD.

Cognitive training (CT) refers to training programs that involve structured practice of specific cognitive tasks with the goal of improving performance in one or more cognitive domains, such as memory, attention, or executive function (13). Playing video games, reading books, practicing writing, remembering spatial locations, remembering objects or words, and communicating more with the patient are some common ways of CT (14–17). Many studies have shown that CT can improve cognitive function (18). Ball et al. (19) found that cognitive function training with three different cognitive functions (memory, reasoning, and processing speed) was effective in improving the cognitive performance in older adults over the age of 65 years, which was maintained for 2 years. Walton et al. (20), Xuefang et al. (21), Hu et al. (22), and Woolf et al. (23) found that CT can improve the cognitive function of patients with Parkinson's disease, stroke, mild cognitive impairment, and major depression, respectively. However, the effects of CT on POCD and POD are controversial. Saleh et al. (14) showed that preoperative CT significantly reduced the incidence of POCD after gastrointestinal surgery. However, other studies have found no significant difference in the incidence of POCD and POD between patients receiving perioperative CT and the control group, and CT had limitations in terms of the feasibility and patient adherence (24, 25). Therefore, in this meta-analysis, we aimed to investigate the effects of CT on POCD and POD.

## 2. Methods

The meta-analysis was conducted in accordance with the Preferred Reporting Items for Systematic Reviews and Meta-analysis (PRISMA) checklist (26). Ethics approval was not necessary because this study was a systematic review and meta-analysis. We registered this study in PROSPERO under number CRD42022371306 (https://www.crd.york.ac.uk/prospero/display_record.php?ID=CRD42022371306).

### 2.1. Search strategy

Two reviewers (Li Zhao and Hongyu Zhu) independently searched PubMed, EMBASE, the Cochrane library, and Web of Science from the inception of the databases to 31 August 2022. The search terms used were as follows: “cognitive training or cognitive intervention or memory training” and “perioperative neurocognitive disorders or postoperative cognitive dysfunction or POCD or postoperative delirium or POD”. No limitation was imposed. In addition, we searched the reference lists of the identified articles for relevant studies and manually screened the additional eligible studies.

### 2.2. Inclusion and exclusion criteria

The inclusion criteria of this study were as follows: (1) Patients in the intervention group received either preoperative CT, postoperative CT, or both. (2) Patients in the control group were treated only for the disease itself, without CT. (3) The diagnostic criteria of POCD and POD were clearly stated in the study. (4) Primary or secondary outcomes must include the incidence of POCD or POD. (5) There was no statistical difference in the cognitive function between the CT and control groups at the time of enrollment. (6) The included studies should be randomized controlled studies or cohort studies. We excluded studies where the data could not be extracted and used for analysis.

### 2.3. Outcomes

The primary outcomes were the incidence of POD and POCD. Secondary outcomes were CT adherence, length of hospital stay, and scores of cognitive function.

### 2.4. Data extraction and assessment of risk of bias

Data extraction and quality assessment were carried out by two independent authors (Li Zhao and Hongyu Zhu). If disagreements arose, they were discussed with the corresponding author (Linji Li). The following information was extracted: first author's name, year of publication, country, the average age of the participants, sample size, types of surgery, type of anesthesia, intervention measures, and results of POCD and POD assessment. Study quality was assessed using the Cochrane risk of bias tool. Some data conversion tools were used to convert interquartile ranges to means and standard deviation in some studies (27).

### 2.5. Statistical analysis

Data analysis was performed by Review Manager (version 5.3) and Stata (version 14) software. Dichotomous and continuous data were analyzed using risk ratio (RR) and mean differences (MD) with 95% confidence interval (CI), with a *P*-value of <0.05 considered statistically significant. Statistical heterogeneity was used to identify the differences among the included studies. *I*^2^ statistic was used to assess statistical heterogeneity, with *I*^2^ > 50% considered to be high heterogeneity and *I*^2^ < 50% considered to be low heterogeneity (28). The random effects model was used if there was high heterogeneity, while the fixed effects model was used if low heterogeneity was detected (29). Sensitivity analyses and subgroup analyses were used for studies with high heterogeneity. Publication bias was measured by Egger's test (30).

## 3. Results

### 3.1. Identification and characteristics of the studies

**Figure 1:**
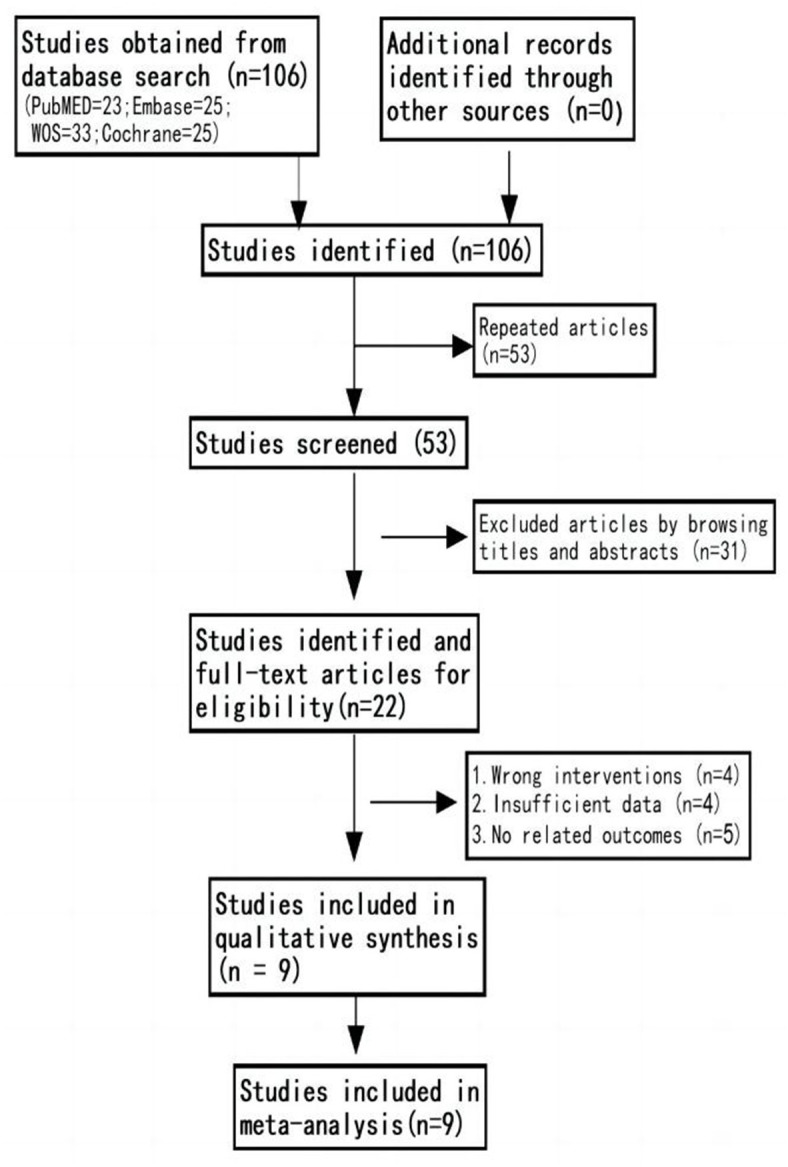
Flow diagram of the literature search strategy.

We initially identified a total of 106 studies through database search. Nine studies were eventually included, with a sample size of 975 cases, including 500 cases in the CT group and 475 cases in the control group (14–17, 24, 25, 31–33). The flow chart of study selection is shown in Figure 1.

**Figure 2:**
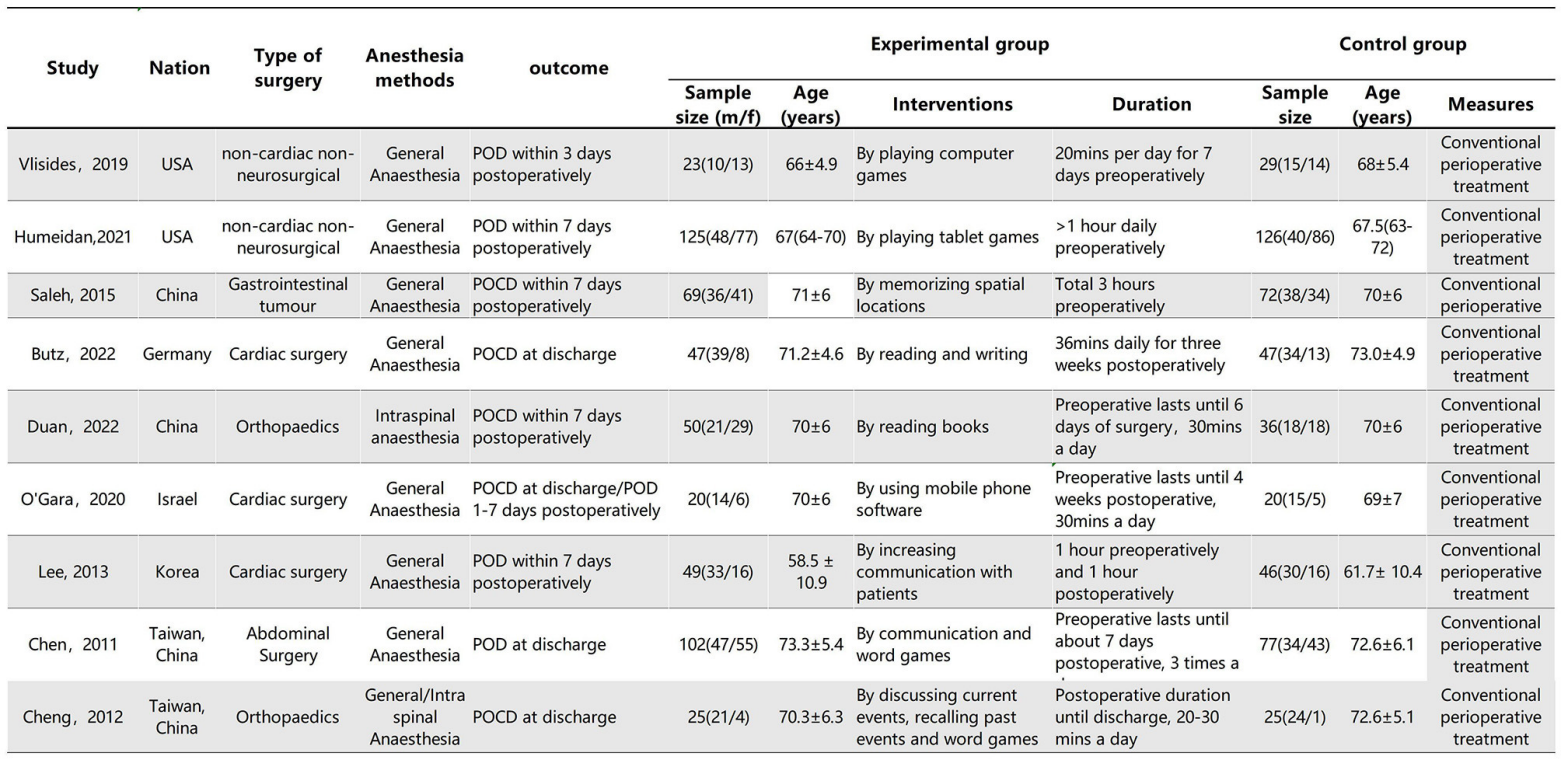
Characteristics of the included studies. Plus-minus values are mean ± SD. IQR, interquartile range.

The characteristics of the studies are shown in Figure 2. A total of five studies assessed the effects of CT on POCD, and five studies assessed the effects of CT on POD.

### 3.2. Quality of the included studies

**Figure 3:**
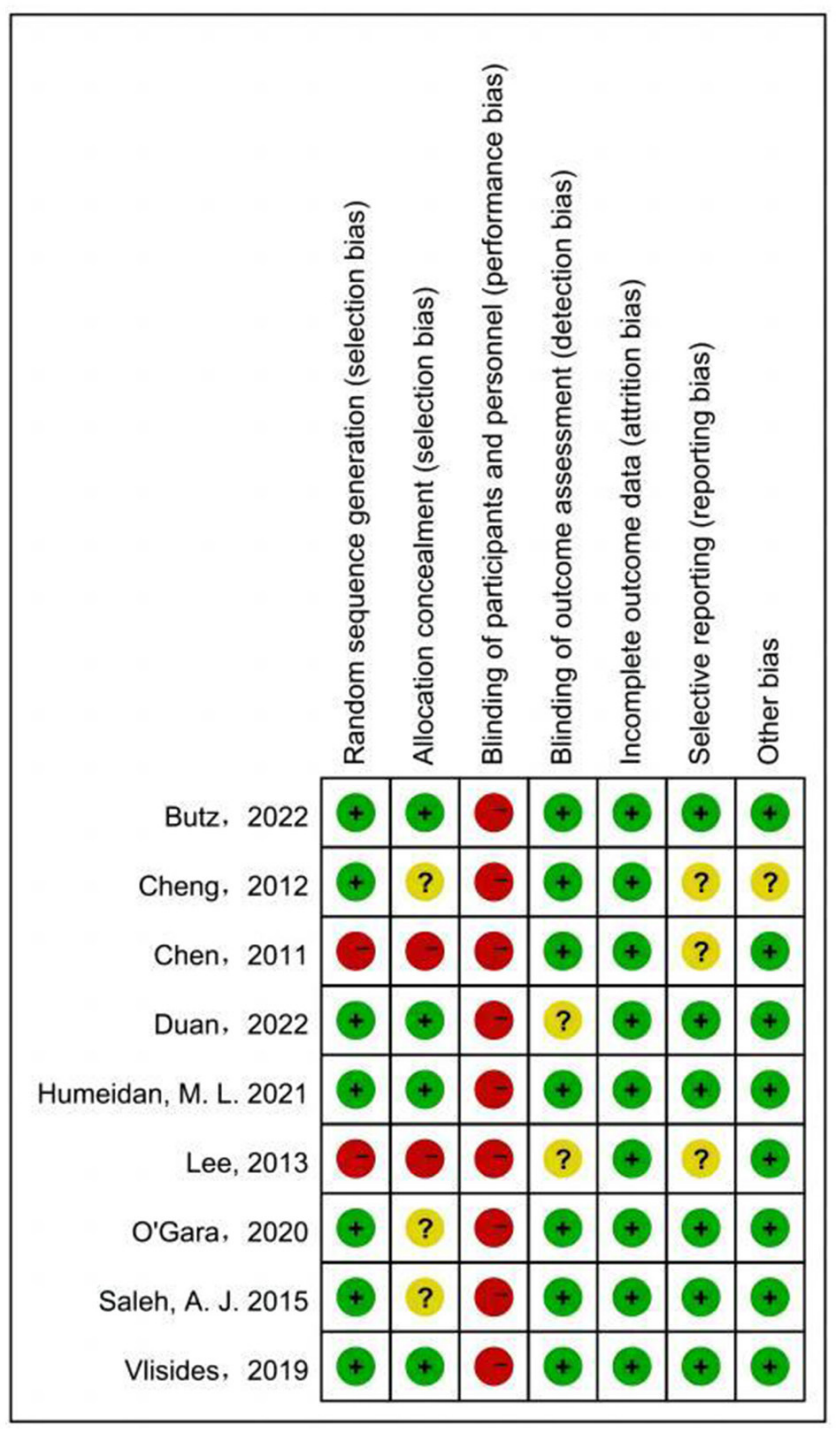
Risk-of-bias summary for all the trials.

The results of assessing the risk of bias for the included studies are shown in the Figure 3. Two studies were considered to have high risk of random sequence generation and allocation concealment (31, 33). Three studies were considered to have unclear risk in allocation concealment (14, 24, 31). None of the included studies were blinded to patients probably because CT requires patient cooperation and takes a long time. Blinding for outcome assessment was unclear in two studies (32, 33). Clinical registrations for three studies were not found, and therefore, the risk was unclear for selective reporting (16, 31, 33). The total sample size of a study was only 50 people, which may have led to partial bias (16).

### 3.3. Primary outcome

#### 3.3.1. Incidence of POCD

**Figure 4:**
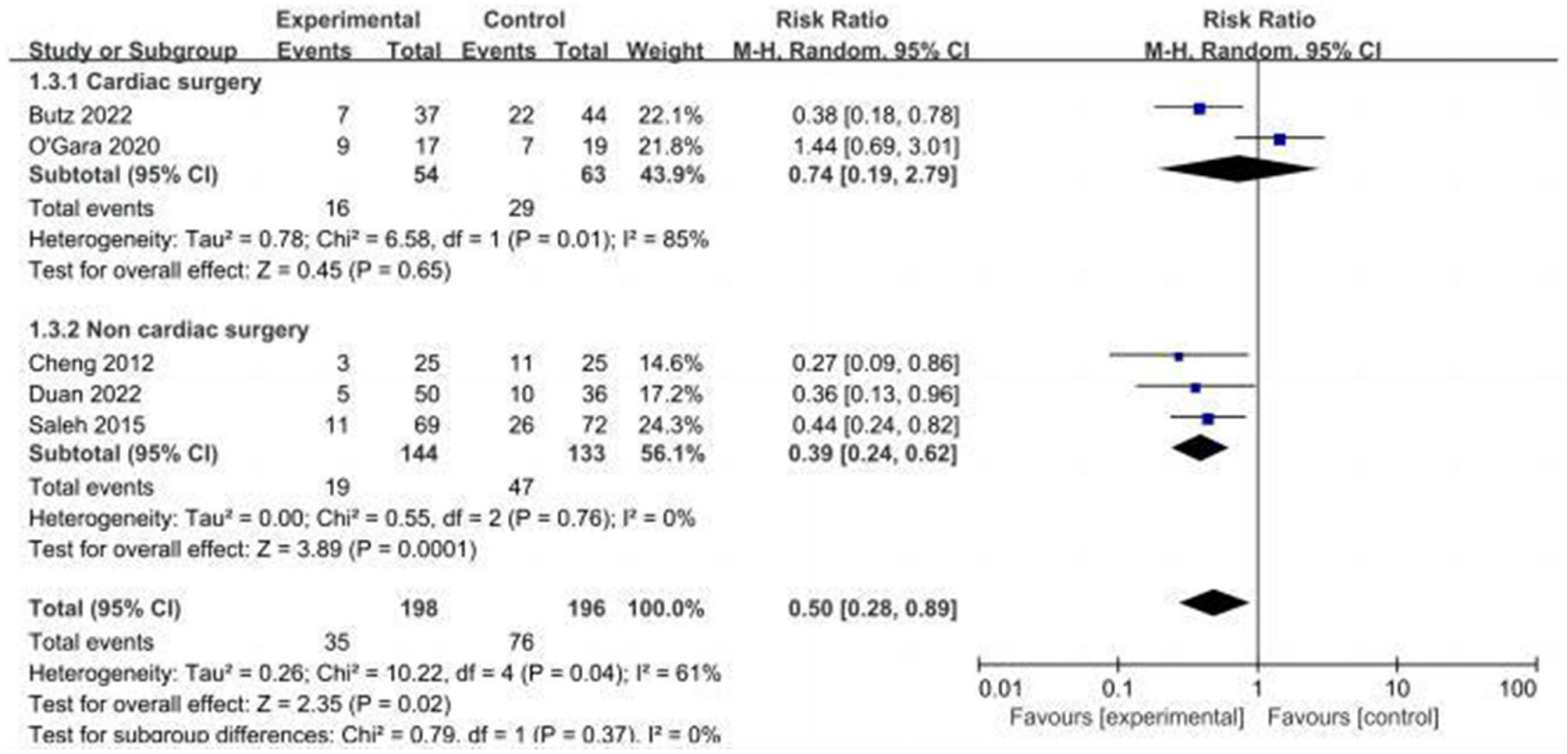
Forest plot of primary outcome—incidence of POCD.

**Figure 5:**
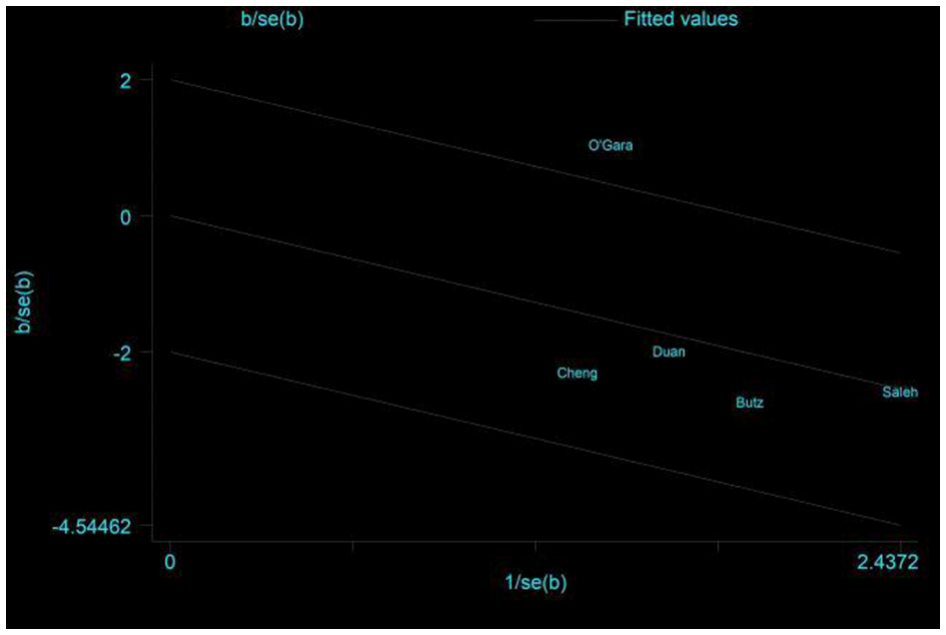
Galbraith plots of primary outcome—incidence of POCD.

**Figure 6:**
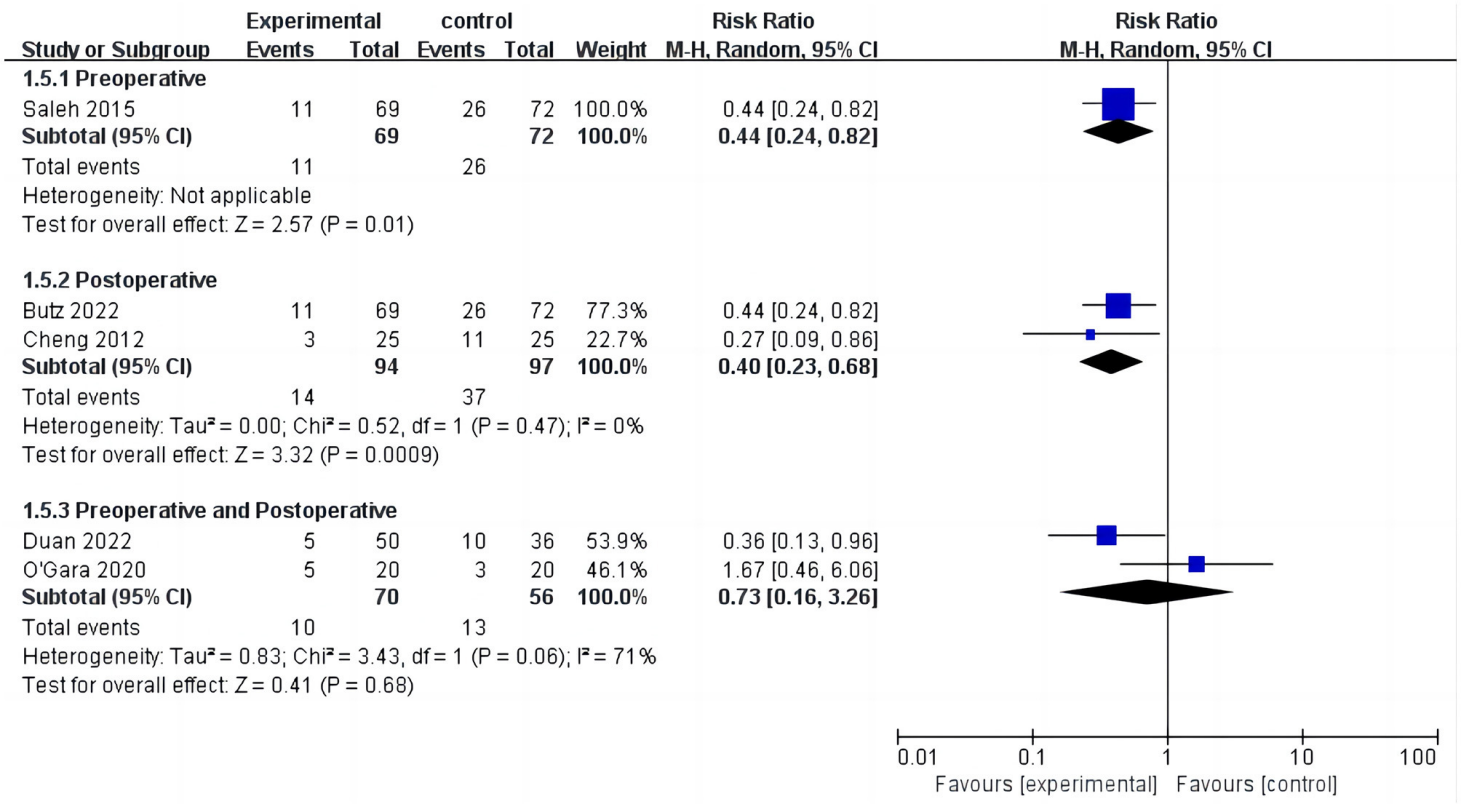
Subgroup analysis of cognitive training timing—incidence of POCD.

Five studies assessed the incidence of POCD (14, 16, 17, 24, 32). Two studies (14, 32) reported the incidence of POCD at 7 days postoperatively, and three studies (16, 17, 24) reported the incidence of POCD at hospital discharge. Due to the high heterogeneity (*I*^2^ = 61%), the random effects model was chosen and showed that the CT group had a significantly reduced incidence of POCD compared to the control group (RR = 0.5, 95% CI: 0.28–0.89, *P* = 0.02, Figure 4). No significant publication bias was found using Egger's test (*P* = 0.718). The Galbraith plots (Figure 5) show a clear heterogeneity between the study by O'Gara et al. and other studies. Sensitivity analysis revealed a significant decrease in heterogeneity (*I*^2^ = 0) when the study by O'Gara et al. was removed, but the result was unchanged (RR = 0.38, 95% CI: 0.14–0.49, *P* < 0.00001). Subgroup analysis (Figure 4) showed significant differences between the CT group and control group for non-cardiac surgery (*I*^2^ = 0%, RR = 0.39, 95%: 0.24–0.62, *P* = 0.0001) but not for cardiac surgery (*I*^2^ = 82%, RR = 0.74, 95% CI: 0.19–2.79, *P* = 0.65). Subgroup analysis of the timing of intervention (Figure 6) revealed that preoperative CT (RR = 0.44, 95% CI: 0.24–0.82, *P* = 0.01) or postoperative CT (RR = 0.41, 95%: 0.26–0.66, *P* = 0.0003) significantly reduced the incidence of POCD, but CT during both preoperative and postoperative periods showed no statistically significant difference compared to the control group (RR = 0.72, 95% CI: 0.17–3.03, *P* = 0.65).

#### 3.3.2. Incidence of POD

**Figure 7:**
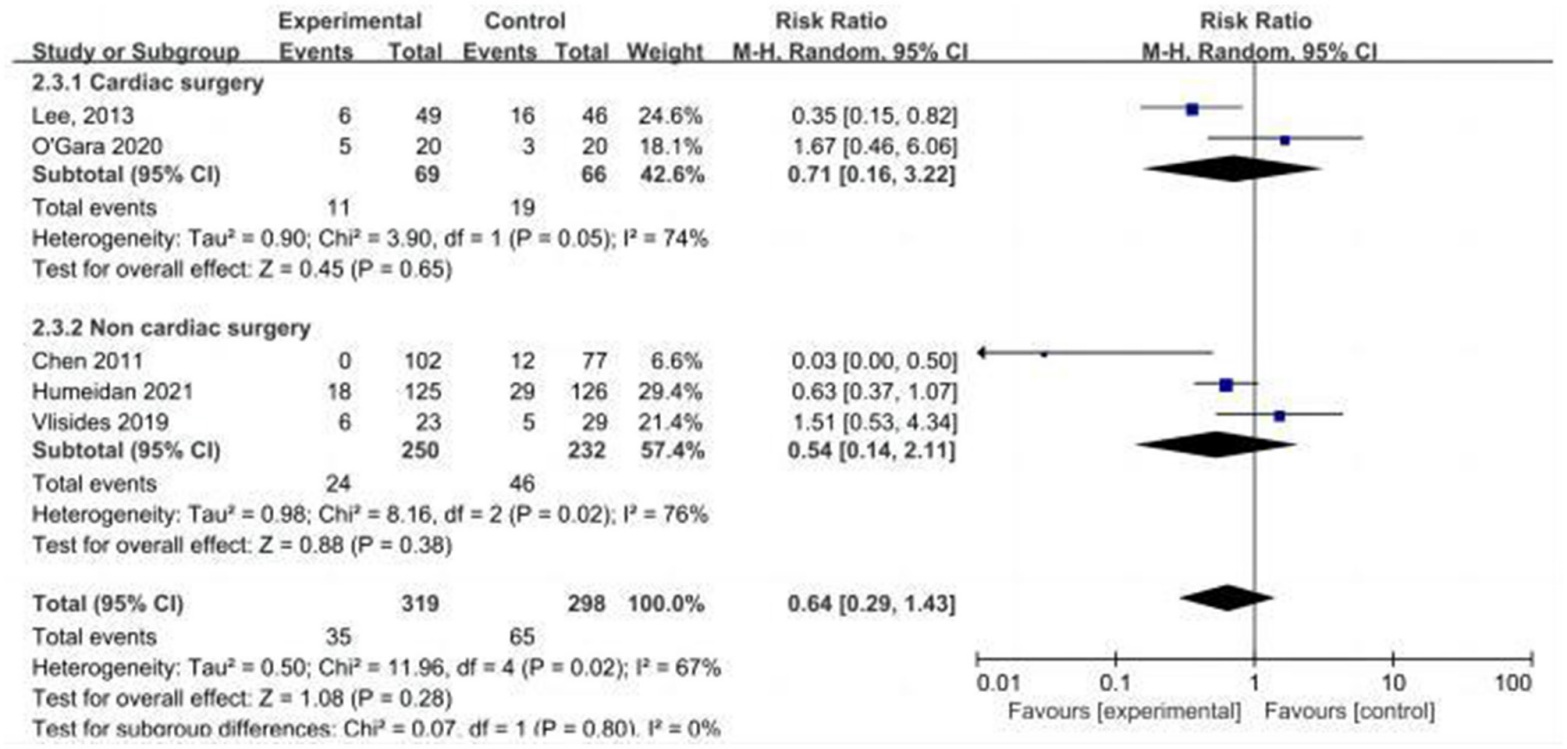
Forest plot of primary outcome—incidence of POD.

**Figure 8:**
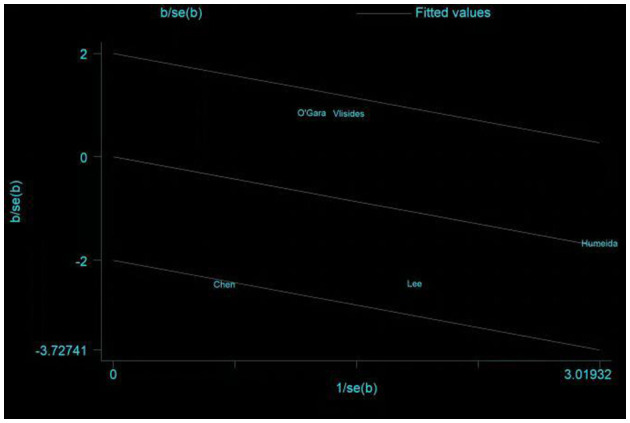
Galbraith plots of primary outcome—incidence of POD.

**Figure 9:**
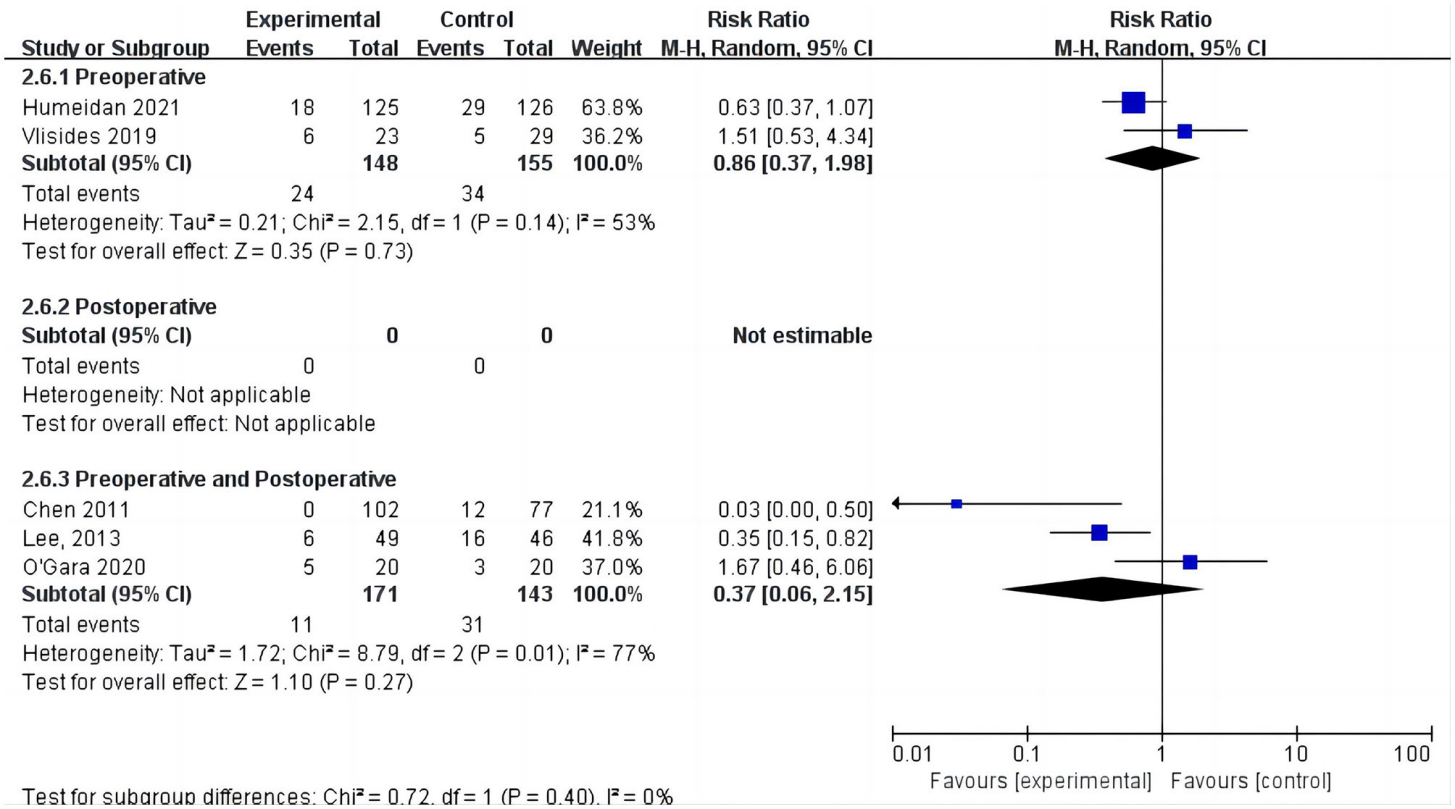
Subgroup analysis of cognitive training timing—incidence of POD.

Five studies assessed the incidence of POD (15, 24, 25, 31, 33). One study (25) reported POD within 3 days postoperatively, three studies (15, 24, 33) reported POD within 7 days postoperatively, and one study (31) reported POD at discharge. Due to the high heterogeneity (*I*^2^ = 67%), we chose the random effects model and the results showed no statistically significant difference between the two groups (RR = 0.64; 95% CI: 0.29–1.43, *P* = 0.28, Figure 7). No significant publication bias was found according to Egger's test (*P* = 0.810). On sensitivity analysis, the results did not change when any of the studies were removed. The Galbraith plots (Figure 8) show a clear heterogeneity between the study by Chen et al. and other studies. The results of the subgroup analysis (Figure 7) showed that there was no statistically significant difference between the CT group and control group for both cardiac surgery (RR = 0.71, 95% CI: 0.16–3.22, *P* = 0.65) and non-cardiac surgery (RR = 0.54, 95% CI: 0.14–2.11, *P* = 0.38). Subgroup analysis of the timing of intervention revealed (Figure 9) that preoperative CT (RR = 0.86, 95% CI: 0.37–1.98, *P* = 0.73) and CT during both the preoperative and postoperative periods (RR = 0.37, 95% CI: 0.06–2.15, *P* = 0.27) were not statistically different compared to the control group.

### 3.4. Secondary outcome

#### 3.4.1. Cognitive training adherence

**Figure 10:**
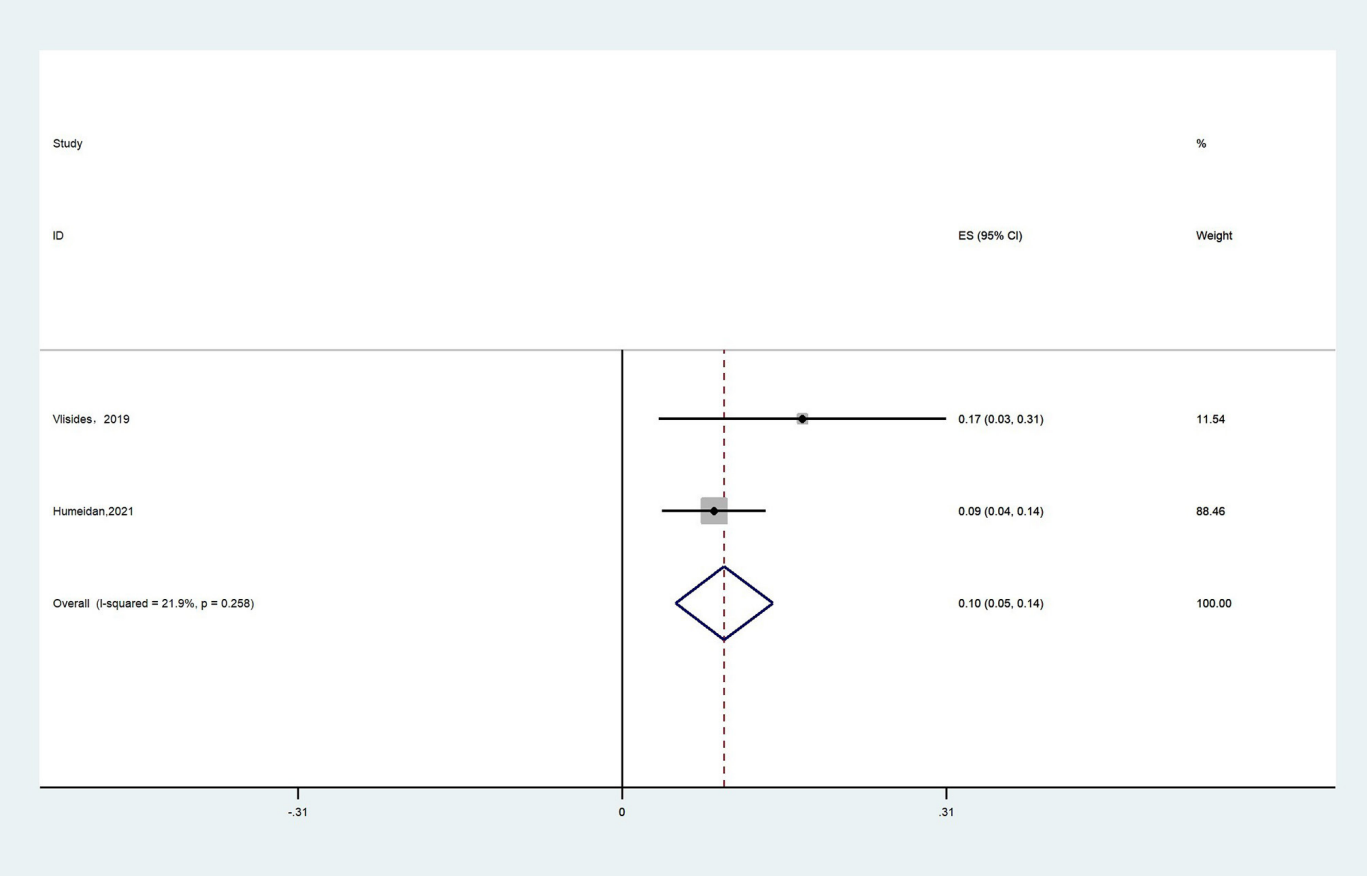
Forest plot of secondary outcome—cognitive training adherence.

Two studies reported CT adherence in the intervention group (15, 25). We defined CT adherence as the proportion of patients in the studies who completed the planned duration of CT. Due to high heterogeneity (*I*^2^ = 21.9%), the fixed effects model was chosen and the result showed that the proportion of patients in the CT group who completed the planned duration of CT was 10% (95% CI: 0.05–0.14, *P* < 0.001, Figure 10).

#### 3.4.2. Scores of cognitive function

**Figure 11:**
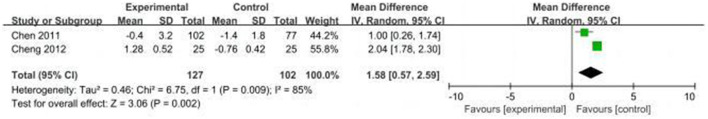
Forest plot of secondary outcome—cognitive function scores.

Two studies (16, 31) used the Mini-mental State Examination (MMSE) scores to assess cognitive function, and one study (24) used the Montreal Cognitive Assessment (MOCA) scores. We extracted the difference by subtracting the baseline measurement from the post-intervention assessment scores of cognitive function in the studies. Due to high heterogeneity (*I*^2^ = 85%), we selected the random effects model. The results showed less decline in MMSE scores in the CT group compared to the control group (MD = 1.58, 95% CI: 0.57–2.59, *P* = 0.002, Figure 11). Another study used MOCA scores, and the difference between the two groups was not statistically significant (*P* = 0.74).

#### 3.4.3. Length of hospital stay

**Figure 12:**
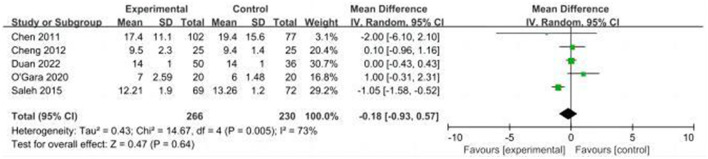
Forest plot of secondary outcome—length of hospital stay.

A total of five studies evaluated the length of hospital stay in the CT and control groups (14, 16, 24, 31, 32). Due to high heterogeneity (*I*^2^ = 73%), we used the random effects model and the results showed that the difference in the length of hospital stay between the CT and control groups was not statistically significant (MD: −0.18, 95% CI: −0.93–0.57, *P* = 0.64, Figure 12). On sensitivity analysis, there was a significant decrease in heterogeneity (*I*^2^ = 1%) when the study by Saleh et al. was removed, but the result was unchanged (MD: 0.08, 95% CI: −0.30–0.46, *P* = 0.68).

## 4. Discussion

With the increasing demand for comfortable perioperative care, more studies have begun to focus on postoperative complications (34). We, therefore, carried out this meta-analysis to evaluate the effect of perioperative CT on POCD and POD. In this meta-analysis, we found that perioperative CT is potentially an effective measure to reduce the incidence of POCD but not the incidence of POD. In addition, our study showed less decline in the cognitive function scores in the CT group compared to the control group. In addition, there was no significant difference in the length of hospital stay. Regarding CT adherence, we found that the proportion of patients in the CT group who completed the planned duration of CT was 10%.

The new 2018 guidelines defined neurocognitive disorders occurring in the perioperative period, including preoperative cognitive impairment, POD, cognitive decline diagnosed within 30 days postoperatively (delayed neurocognitive recovery), and cognitive decline diagnosed within 2–12 months postoperatively (35). As most previous studies have used POD and POCD as the outcome indicators of postoperative cognitive function, we also used POCD and POD to assess the postoperative cognitive function (4).

This meta-analysis showed that perioperative CT significantly reduced the incidence of POCD (*P* = 0.02). Possible mechanisms underlying the effects of CT in improving cognitive function are as follows. First, CT may increase the density of cortical dopamine D1 receptors, which play a key role in human cognition as it is dependent on adequate dopamine neurotransmission (36). Second, Feinkohl et al. found that patients with more cognitive reserve had a lower incidence of POCD (36). In addition, Mondini et al. have found that CT enhances patients' cognitive reserve (37); thus, CT may improve patients' cognitive function by enhancing their cognitive reserve. Third, studies have found that cognitive function, perception, and memory function decline progressively with age, but the brain retains lifelong plasticity and adaptive reorganization; therefore, some cognitive functions of the brain can be improved by using appropriately designed training programs (38–40).

Furthermore, the results of this meta-analysis showed no statistically significant difference in the incidence of POD between the CT and control groups (*P* = 0.28). The reason for this outcome is unclear, and it is speculated that it may be due to significant heterogeneity (*I*^2^ = 67%) and inadequate sample size of the study.

Of note, three studies assessed CT adherence in the CT group (15, 24, 25). The proportion of patients in the CT group who completed the planned duration of CT was 10% (15, 25). One study reported that the main reasons for low CT adherence were lack of computer access, time constraints, and feeling overwhelmed (25). Another study reported that the main reasons were “I did not have enough energy, I forget, the frequency of game was too often (24).” Therefore, simplifying the training methods and providing computer assistance are necessary to avoid low adherence. O'Gara et al. found that a low proportion of people completed the total scheduled training duration (10 h); however, most were able to complete a longer duration of the cognitive training (>4 h).

Statistical heterogeneity was high in our meta-analysis. This heterogeneity may be due to differences in the types of surgery, diagnostic tools for cognitive function, mean age, duration of CT, timing of CT, and methods of CT. However, we performed subgroup analyses for the type of surgery and timing of CT. Only the non-cardiac surgery subgroup for the POCD outcome showed low heterogeneity (*I*^2^ = 0). Subgroup analyses for other categories were not performed due to the variety of diagnostic tools for cognitive function and CT methods, lack of detailed intervention duration, and the small difference in mean age.

## 5. Limitations

There are some limitations of this study. First, the sample size was relatively small. Second, many factors including the methods of CT, duration of CT, and diagnostic methods for POCD and POD differed among the studies, which led to high clinical heterogeneity. Third, as the incidence rates of POCD and POD were our primary outcomes, we excluded studies that did not include data on POCD and POD; therefore, the evidence for secondary outcomes may be insufficient.

## 6. Conclusion

Our meta-analysis revealed that perioperative cognitive training is possibly an effective measure to reduce the incidence of POCD but not for the incidence of POD.

## Data availability statement

The original contributions presented in the study are included in the article/Supplementary material, further inquiries can be directed to the corresponding author.

## Author contributions

LZ designed the study and wrote the manuscript. LZ, HZ, XZ, and WM participated in the extraction and analysis of the data. LL and YX critically supervised, evaluated, and validated the article. All of the authors worked on the article and agreed with the submitted version.
