# Supplementary material for: Effects of perioperative cognitive function training on postoperative cognitive dysfunction and postoperative delirium: a systematic review and meta-analysis
Source: Front Neurol. 2023 Jun 21;14:1146164. doi: 10.3389/fneur.2023.1146164 (PMC10322196; doi:10.3389/fneur.2023.1146164)
Supplement: Supplementary file 1 [file Table_1.DOCX]

| **Database** | **Search strategy** | **Number** |
| --- | --- | --- |
| **PubMed** | (((((((((Cognitive Dysfunction*[Title/Abstract]) OR (Cognitive Impairment*[Title/Abstract])) OR (Cognitive Disorder*[Title/Abstract])) OR (Neurocognitive Disorder*[Title/Abstract])) OR (Cognitive Decline*[Title/Abstract])) OR (Mental Deterioration*[Title/Abstract])) OR (deliri*[Title/Abstract])) AND ((Postoperative*[Title/Abstract]) OR (post-operative[Title/Abstract]))) OR (((POD[Title/Abstract]) OR (POCD[Title/Abstract])) OR (Perioperative neurocognitive dysfunction[Title/Abstract]))) AND (((((((((cognitive therapy[Title/Abstract]) OR (cognitive training[Title/Abstract])) OR (cognitive retraining[Title/Abstract])) Perioperative neurocognitive dysfunctionOR (cognitive support[Title/Abstract])) OR (cognitive stimulation[Title/Abstract])) OR (cognitive intervention[Title/Abstract])) OR (cognitive exercise[Title/Abstract])) OR (memory therapy[Title/Abstract])) OR (memory training[Title/Abstract])) | 23 |
| **Web of Science** | **(TS=(“cognitive therapy” OR “cognitive training” OR “cognitive retraining” OR “cognitive support” OR “cognitive stimulation” OR “cognitive intervention” OR “cognitive exercise” OR “memory therapy OR memory training” )) AND ((TS=(“deliri*” OR “Cognitive Dysfunction*” OR “Cognitive Impairment*” OR “Cognitive Disorder*” OR “Neurocognitive Disorder*” OR “Cognitive Decline*” OR “Mental Deterioration*” ) AND (TS=("Postoperative*" OR "post-operative"))) OR (TS=("Perioperative neurocognitive dysfunction" OR "POCD" OR "POD")))** | 33 |
| **Embase** | ('cognitive therapy':ab,ti OR 'cognitive training':ab,ti OR 'cognitive retraining':ab,ti OR 'cognitive support':ab,ti OR 'cognitive stimulation':ab,ti OR 'cognitive intervention':ab,ti OR 'cognitive exercise':ab,ti OR 'memory therapy':ab,ti OR 'memory training':ab,ti) AND ((('deliri*':ab,ti OR 'Cognitive Dysfunction*':ab,ti OR 'Cognitive Impairment*':ab,ti OR 'Cognitive Disorder*':ab,ti OR 'Neurocognitive Disorder*':ab,ti OR 'Cognitive Decline*':ab,ti OR 'Mental Deterioration*':ab,ti) AND ('Postoperative*':ab,ti OR 'post-operative':ab,ti)) OR 'Perioperative neurocognitive dysfunction':ab,ti OR 'POCD':ab,ti OR 'POD':ab,ti ) | 25 |
| **Cochrane** | #1 “cognitive therapy” OR “cognitive training” OR “cognitive retraining” OR “cognitive support” OR “cognitive stimulation” OR “cognitive intervention” OR “cognitive exercise” OR “memory therapy OR memory training” 10742  #2 “deliri*” OR “Cognitive Dysfunction*” OR “Cognitive Impairment*” OR “Cognitive Disorder*” OR “Neurocognitive Disorder*” OR “Cognitive Decline*” OR “Mental Deterioration*” 15339  #3 "Postoperative*" OR "post-operative" 135118  #4 "Perioperative neurocognitive dysfunction" OR "POCD" OR "POD" 2965  #5 (#1) AND ((#2 AND #3) OR #4) 69  #6 only [Trials](http://www-cochranelibrary-com-443.webvpn.bjmu.tsg211.com/en/search?searchBy=1&searchText=PEEP&isWordVariations=&resultPerPage=25&searchType=basic&forceTypeSelection=true&selectedType=central&displayText=&orderBy=relevancy&p_p_id=scolarissearchresultsportlet_WAR_scolarissearchresults&p_p_lifecycle=0&p_p_state=normal&p_p_mode=view&p_p_col_id=column-1&p_p_col_count=1) 25 | 25 |
